# Semi-flipped classroom-based learning interventions in a traditional curriculum of oral medicine: students’ perceptions and teaching achievements

**DOI:** 10.1186/s12909-023-04017-6

**Published:** 2023-01-19

**Authors:** Yun Hong, Jiaying Wu, Jie Wu, Huaimin Xu, Xiaolan Li, Zhengmei Lin, Juan Xia

**Affiliations:** Guanghua School of Stomatology, Hospital of Stomatology, Sun Yat-Sen University, Guangzhou, 510055 Guangdong China

**Keywords:** Oral medicine, Advanced dental education, Massive open online course, Semi-flipped classroom, Oral mucosa disease, Online education

## Abstract

**Background:**

In recent years, flipped classes have emerged and become popular in college medical education. However, due to the huge medical learning system and the limited pre-class study time of students, it is difficult to implement in all courses. And then we adopted the semi-flipped classes (SFCs) to evaluate its teaching effect. This study analysed three educational methods that can be used in oral medicine courses: online education, offline education, and semi-flipped classes.

**Methods:**

We used two surveys to evaluate the three educational methods. In the first survey 46 teachers and 238 undergraduates shared their experience of the live-streaming and traditional offline courses offered in the different oral medicine curricula; we used anonymous questionnaires to evaluate their class experience. In the second survey 94 students shared their experience of the semi-flipped and traditional classrooms. Students who attended the SFCs in the experimental group learned about the oral mucosa disease by themselves using an online video course and then participated in offline interaction with teachers. The evaluation of the above educational methods was conducted using the anonymous questionnaires and final exam assessment.

**Results:**

According to the first survey, teachers and students both agreed that the overall teaching experience and learning effectiveness in offline education are superior to those in online education. According to the second survey, students who participated in the SFCs performed better in the final exam than those who participated in the simple offline classes. Additionally, the survey showed that the new teaching method helped students gain more knowledge and positively influenced their clinical practice.

**Conclusions:**

Compared with the online and offline educational methods, the SFC showed better results in both the questionnaire and final exam assessment. Hence, the effectiveness of medical education can be improved by adopting a teaching mode that combines online and offline teaching methods. Scientific and logical SFCs designs, along with their effective implementation, would eventually make SFCs an important tool for medical education.

## Background

Traditional offline teaching approaches require students to listen to teachers’ lectures and complete homework after class. This strategy is not good for students’ imagination, initiative, and knowledge attainment to some extent [1]. And how to deliver a large amount of information most effectively is a question that has long plagued educators [2]. However, with the rapid development of the internet, the learning style of students has changed, and educators are able to utilise appropriate teaching modes, for example, online video open classes, e-learning courses, and even massive open online courses (MOOCs). The common feature of these examples is that they facilitate learning by overcoming the limitations of time and space [3].

Since J. Wesley Baker first proposed the ‘classroom flip’ at the 11^th^ International Conference on College Teaching and Learning in 2000 [4], this teaching strategy has been applied to primary and secondary education worldwide. In flipped classrooms, which combine online and offline educational methods, students watch online lectures, participate in online discussions, or carry out research at home, while actively engaging with concepts in the classroom with a mentor's guidance [5]. In this way, students have opportunities to find information quickly and take the initiative in learning. Usually, the teaching sessions are based on video courses with clear and appropriate information, which can be reviewed at any time [6].

Educators have begun to gradually explore the application of flipped classrooms in college education [7]. In this study, we estimated three educational methods for a general oral medicine course: online education, offline education, and semi-flipped classes (SFCs), with the aim to potentially reform college educators’ teaching methods and found a suitable learning style for our medical students. The analysis showed the existing basic online and offline course managements, which represent the teacher-led mode. However, compared with the traditional offline course, the online teaching method makes it more difficult for lecturers to get feedback, given that interactions do not take place face to face. Furthermore, owing to the complexity of medical education, applying the flipped classroom was difficult. Therefore, we aimed to investigate a combination based on MOOCs and flipped classes: a SFC. Accordingly, we developed an online–offline interacting SFC for the oral mucosa disease course, which typically deals with stomatology and is concerned with the diagnosis and therapy of the related diseases [8]. It usually represents the interplay between physical conditions and oral health [9]. By using the SFC approach, we expect to build a flexible and enthusiastic classroom, in which students actively ask questions and even play the dominant role in the class. Through this student-led teaching method, we aim to recommend a better teaching approach for medical education.

## Methods

### Traditional course management

According to the course’s established training plan and syllabus, we developed two curricula: offline education and online education, with no significant difference in teaching time, frequency, and duration. Generally, traditional offline courses take place in a classroom through face-to-face teaching with PowerPoint and the necessary teaching aids. The college requires all teaching and research departments to conduct collective lesson preparation. In this study, we offered all oral medicine courses through offline education. Teaching experts carried out inspections regularly and reported their feedback to the education department. They collect feedback by observing courses and filling out evaluation forms on teachers' teaching, such as interaction with students, explanation professional, and multimedia application.

### Online live-streaming course management

We aimed to create a sense of presence in the online live-streaming course by using the innovative ‘on-site classroom-style’ live-broadcast method. Instructors were required to go back to school, enter the classroom, and step onto the podium to restore the traditional offline teaching environment. In this study, we also provided all oral medical courses through online education. Each class was broadcast live in a lecture hall of the college, which was equipped with professional facilities; a teaching assistant was assigned to the teaching site or platform to supervise the students' attendance and learning effectiveness.

### Semi-flipped course management

To achieve the combination of a MOOC and flipped class and the reformation of the oral mucosa curricula, we designed and evaluated an online SFC course [10]. In this curriculum, students receive their studying materials at their own pace before the class starting. These studying materials include some typical cases and tasks (Table 1). We designed the SFC course which is closely related to the previous content. In the class, the teaching content will revolve around these pre-class learning materials. These methods encourage students to lead the class by using the cases or questions given to them before the class.

**Table 1:** Description of the course design

	Oral bullous diseases	Oral ulcerous diseases	Oral patches stria diseases
Learning targets / course outline	1. To get familiar with cicatricial pemphigoid	1. To provide an overview of oral ulcerous diseases	1. To get familiar with oral potentially malignant disorders and precancerous conditions
	2. To develop a deep understanding of pemphigus	2. To develop a basic understanding of Behcet’s disease and traumatic ulcers	2. To develop a deep understanding of oral lichen planus, oral leukoplakia, erythroplakia, discoid lupus erythematosus, and oral submucous fibrosis
	3. To enhance the ability to distinguish and diagnose oral bullous diseases	3. To get familiar with the classification, clinical feature, diagnosis, and treatment of recurrent aphthous ulcers	3. To develop the ability to differentiate between oral patches stria diseases
Learning difficulties	Differentiating between the diagnosis of pemphigus and diagnoses other erosive lesions	Distinguishing between the cause of oral ulcers and provide appropriate treatments	1. Diagnosing various diseases with white changes
			2. Understanding the lesion characteristics of oral lichen planusbetween the different positions
			3. Accurately identifying oral diseases with white lesions
Course design	Provide cases before class to exhibit the diagnosis process of oral bullous diseases, based on several clinical cases, in gradual difficulty	Task-driven teaching method	Provide pre-class problems and situational teaching method
Implementation	When reporting clinical cases, lecturers shared their clinical thinking and gradually solved the mystery of the disease. Several in-class questions about key nodes of the diagnosis process were interspersed	To inspire students’ self-directed learning, lecturers gave a presentation on Behcet’s disease in the video course, as an example, and assigned students a task about the aetiology of the different types of ulcers. The in-class time was used to communicate what students learned	An auction was held during class time, and the knowledge beyond the textbook was taken as the subject matter. In this fictitious auction, students bided for the knowledge / questions in teams

We adopted the clinical cases to exhibit the diagnosis process by providing an oral bullous disease course. Accordingly, students could learn how to make basic diagnoses from complex conditions [11]. Students analysed a clinical case in a group before the class. In the class, under the guidance of teachers, students were encouraged to provide a diagnosis with a reasonable explanation, and actively contact more cases for comparative study. The method is more conducive to cultivating learners’ team spirit and ability to analyse practical problems logically.

This task-driven teaching method trained students’ independent learning ability [12, 13]. Before the class, students were assigned a task about the aetiology of the different types of ulcers. Students were required to give a small presentation on Behcet’s disease (‘oral ulcerous diseases’) in class and share what they learned during group work.

We designed the ‘oral patches stria diseases’ course. In this method, students were required to watch a video clip to preview the relevant chapters before the class, and the knowledge beyond the textbook was taken as the subject matter. At the same time, some relevant problems will be given to students to guide their thinking. In the class, students answered questions about the course and were inspired to ask more questions by thinking about the problems before class. This change allowed students to learn to explore and solve problems [14–16].

Often, the clinical situation was found to be too complicated; therefore, the clinical experience could only be achieved through face-to-face interactions. We designed the SFC course to help students effectively establish clinical thinking and master clinical skills [17, 18].

### Survey of the online and offline educational methods

In the surveys, 46 teachers who all had traditional offline teaching experience, took part in three case studies. Each teacher had two lessons, a total of 90 min, taught the same lesson using the same methods. The teachers who taught the oral mucosa courses also participated in the SFCs. Two hundred and thirty-eight students participated in this survey of the online education from grade 2016 (i.e., year 4 students graduating in 2021) and offline education from grade 2017 (i.e., year 3 students graduating in 2022).

Ninety-four students, from grade 2015 (i.e., year 5 students graduating in 2020), participated in the survey of the SFCs. The control group (grade 2017) was composed of former students enrolled in the traditional teaching method (Table 2).

**Table 2:** Characteristics of the students

	Grade 2015(*n* = 94)	Grade 2016(*n* = 152)	Grade 2017(*n* = 86)
Gender, *n*			
Male	31	50	28
Female	63	102	58
Student Category			
Domestic	87	140	77
International	7	12	9
College Entrance Examination (Full Score = 750)	610 ± 20	600 ± 20	605 ± 20

### Questionnaire and subjective evaluation

After completing the courses, the students and teachers were required to fill out detailed questionnaires (Tables 3 and 4). To evaluate the outcomes of the SFC method, the post-course survey was split into two phases. The first phase sought to analyse students’ subjective evaluations of video-based courses in SFCs. The second phase was a statistical analysis to estimate the effects of this teaching method on the objective final exam result.

**Table 3:** Questionnaire for students: Teaching quality of traditional offline courses and online live-streaming courses

This questionnaire mainly investigates the advantages and disadvantages of the current online live courses and traditional offline courses, with the aim to improve overall educational methods. Please check the following assessment items and then answer or score (1–10) truthfully (the higher the evaluation, the higher the score)	
Before the class	1. Please rate your enthusiasm in lessons
	2. Please rate your comfort and convenience level in lessons
	3. Please rate your satisfaction with the acquisition of courseware and other learning materials before class
	4. Please rate your concentration level in lessons
	5. Please rate the interference level of the external environment in lessons
	6. Please rate the clarity of the teacher's drawings in lessons
	7. Please rate the clarity of the teacher’s demonstrations in lessons
	8. Please rate the learning effectiveness of the blackboard writing in lessons
	9. Please rate your level of enthusiasm to answer questions in lessons
	10. Can the questions during lessons be fed back to the teacher in a timely and effective manner
	11. Please rate the promptness of teachers' answers and instructions after class
	12. Please rate the convenience level of reviewing the knowledge of the course/after-school review
	13. According to your interests/preferences, score the different learning modes
Effectiveness of learning	1. Please rate the interaction effect in lessons
	2. Correctness of self-test questions and classwork
	3. Please rate the after-class review efficiency
	4. Is recording and playback of online courses useful for learning
	5. Overall learning effectiveness evaluation
	6. Please list the reasons influencing your learning effectiveness evaluation
Evaluation of teachers	1. Is the teaching content intensively combined with the syllabus
	2. Please rate the teacher’s attention level to the course
	3. Please rate the enrichment of the course content prepared by the teacher
	4. Did the teacher speak clearly and at a moderate rate in class
	5. Was the PowerPoint courseware clear and attractive in teaching
	6. Did the teacher provide sufficient information in class
	7. Did the teacher make students fully understand the key and difficult points of the course content
	8. Did the teacher organize, manage, and control the class well
	9. Did the teacher pay attention to teaching methods
Aspect of course	1. Which mode do you think is more suitable for the theoretical course this semester
	2. What is your favourite course among the online live-streaming courses
Satisfaction level of the education department’s work	1. Please rate your satisfaction level of the course schedule
	2. Please rate your satisfaction level of class guidelines
	3. Please rate your satisfaction level of effective problem solving
	4. Please rate your overall satisfaction level of the live teaching of our school
	5. Please list the difficulties you encountered in the online live-streaming courses
	6. Has the learning experience of this live course inspired you to improve your learning methods? If yes, please describe how you would like to adjust this in the future
	7. If you have other suggestions or comments, please list them

**Table 4:** Questionnaire for teachers: Teaching quality of traditional offline courses and online live-streaming courses

This questionnaire mainly investigates the advantages and disadvantages of the current online live-streaming courses and traditional offline courses, with the aim to improve overall educational methods. Please check the following assessment items and then answer or score (1–10) truthfully (the higher the evaluation, the higher the score)	
Before the class	1. Enthusiasm of teaching theoretical courses
	2. Attention level to the online course of your education department
	3. Average preparation time for the two-hour lesson
	4. Which aspect did you focus on when preparing for the lesson?
	5. Scrupulousness about the medical records leakage
	6. Whether to give materials or courseware to the students efore class for preview and review
	7. Whether to arrange quizzes and questions in class or homework after class
In class	1. Can youquickly adapt to the classroom environment and teaching methods
	2. Please rate your lecture performance and passion
	3. Please rate the operating convenience level of classroom facilities and equipment
	4. Please rate the frequency of teacher and student interaction
	5. Please rate the convenience level of teacher and student interaction
	6. Please rate the accuracy of the students' answers to questions in class
	7. Please rate the diligence level of students in class
	8. Please rate the convenience level of reforming the teaching methods
After class	1. Please rate the satisfaction with the teaching effectiveness
	2. Please rate the different teaching methods (i.e., online live-streaming and traditional offline teaching) on the same course
	3. Please rate the different teaching methods on the cultivation of students' independent learning ability
	4. Overall, which teaching method is suitable for this course in your opinion?
	5. Please rate the teaching management department
	6. What are the difficulties you encountered during the online live-streaming teaching process? Please put forward any suggestions for improvement
	7. What assistance do you need the most in the process of preparing lessons, in developing courseware, and during class
	8. Has this online live-streaming teaching experience contributed to your ability to reform your teaching methods? How do you hope to reform your teaching methods in the future?
	9. If you have other suggestions or comments, please list them

#### Data collection and statistical approach

This study evaluating teachers' feedback between online and offline teaching methods belongs to the fixed design of the paired design. First, it is judged on whether it meets the applicable conditions of the paired design t-test, that is, whether the difference comes from the normal distribution. By performing a normality test on the difference, it was found that it is from the normal population (W-test *P* < 0.05); finally, the paired design t-test signed ranks sum test was used for statistical analysis. This study evaluating students' feedback between online and offline teaching methods belongs to t-test.

### Data collection of the final examination

The final exam was a closed-book exam covering all course sections. To ensure the impartiality of the exam questions, the questions were set independently by teachers who did not participate in the SFCs. The total score of the exam paper was 30 points. The standard score (SS) was calculated with the formula SS = OS × DF, where OS is the original score on the test paper and DF is the difficulty factor, which was computed according to the requirements of the syllabus. For example, the difficulty coefficient of the knowledge points that represented the grasping / mastering of the syllabus was 0.9, while those knowledge points that represented familiarity or understanding were 1.1 and 1.2, respectively. All results were weighted to obtain the difficulty factor of each test paper.

## Results

### Teachers and students' feedback on online and offline courses

**Fig. 1:**
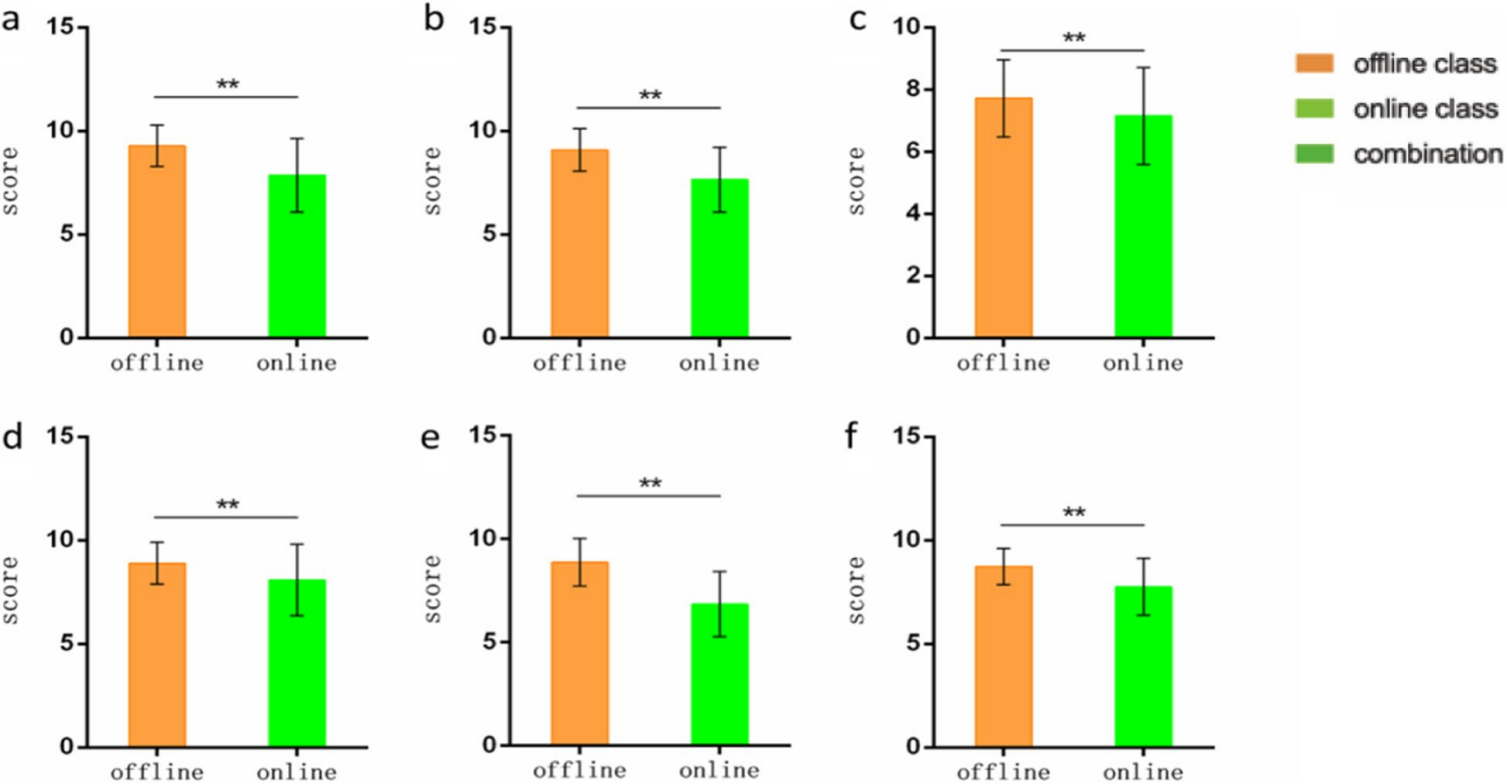
Teachers' evaluation results of online teaching are lower than that of offline one. **a**, Adaptation to the classroom environment and teaching methods. **b**, Expressiveness, and passion during the lecture. **c**, The accuracy of students answering questions. **d**, Operational convenience of classroom equipment and facilities. e, Teacher-student communication frequency. f, Satisfaction with teaching effect. (**p < 0.01)

Although the teachers had prepared more intensely for online teaching than for offline teaching and carried out a wide range of inspections and supervision, which significantly improved the quality of teaching, the result was not as expected. According to the feedback after classes, which covered teachers’ adaptations to the online live-streaming lessons; teachers’ expressiveness and passion during lectures; students' accuracy in answering questions; the convenience of operating classroom equipment and facilities; teacher–student interaction frequency; and overall satisfaction with the teaching effectiveness (Fig. 1), the teachers’ ratings of online courses, on average, were lower than those of traditional offline courses.

**Fig. 2:**
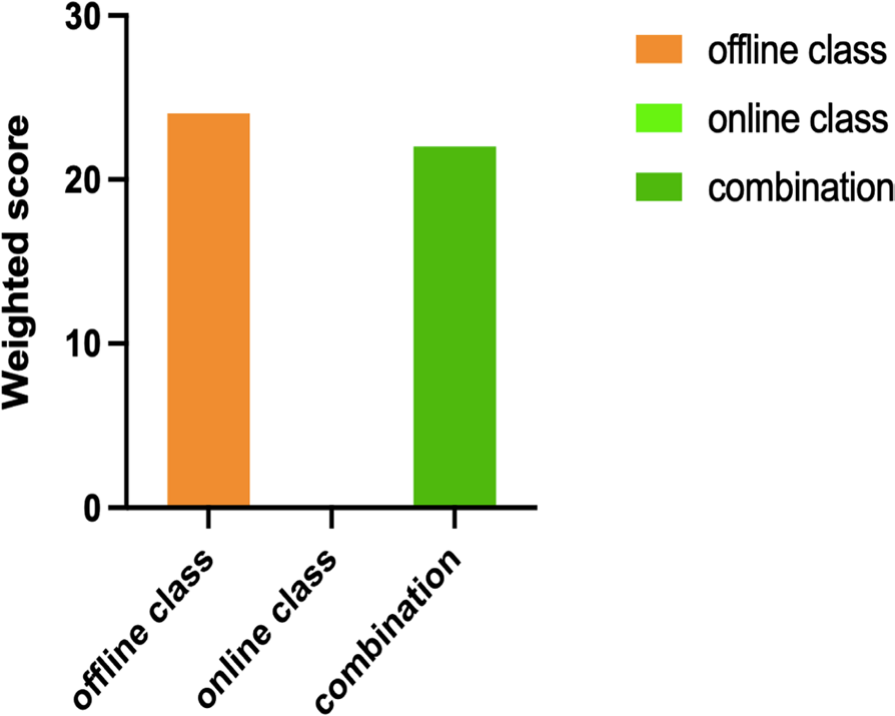
Teachers' evaluation on more suitable method for teaching

Of the 46 teachers surveyed, 24 (52.2%) believed that the offline courses were more suitable; 22 (47.8%) believed that mixed online and offline teaching modes were better; and no teachers believed that the courses conducted via online live-streaming classes only were effective (Fig. 2).

**Fig. 3:**
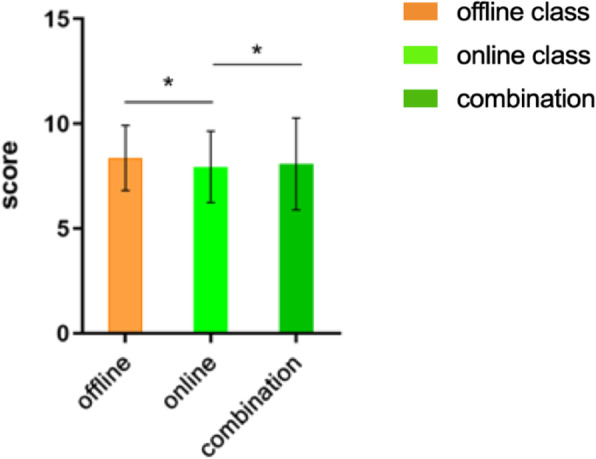
Students' evaluation on different kinds of courses. (**p < 0.01)

The students also believed that online courses were not as effective as traditional offline courses in terms of classroom interaction, efficiency of after-class reviews, access to the course’s information, and the teacher’s organisation and control of the class. The overall learning effectiveness of traditional offline courses was much better according to the students’ feedback (Fig. 3). Therefore, they were more inclined to choose traditional offline courses or a combination of online and offline education.

### Questionnaire for semi-flipped classes

#### Overall evaluation

**Fig. 4:**
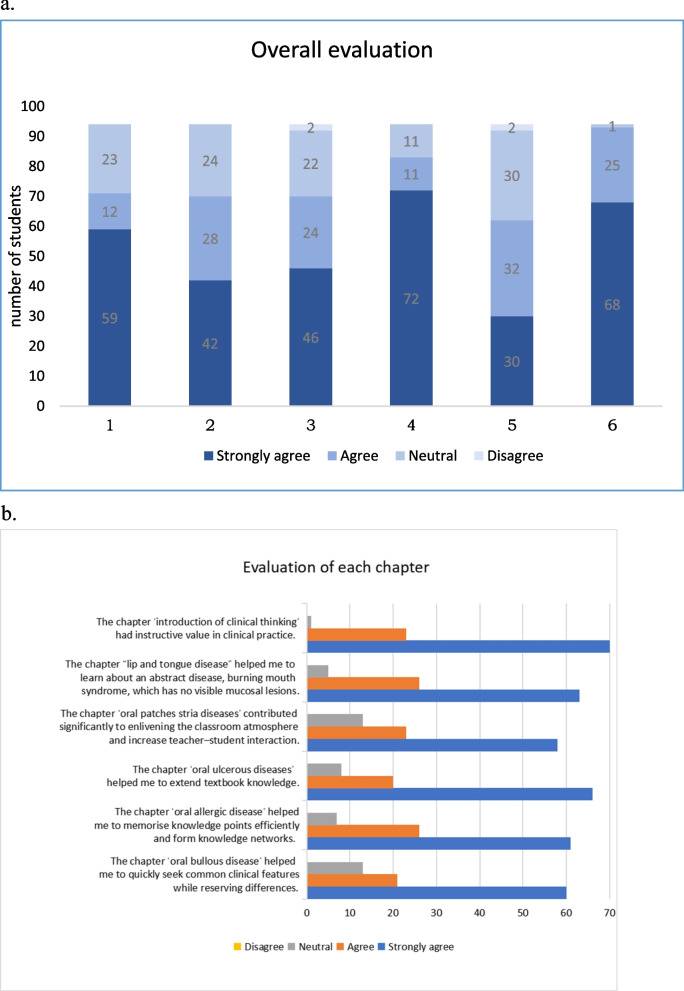
Statistics Questionnaire for Semi-Flipped Classes. **a**, Overall evaluation of SFCs (the abscissa 1–6 refers to the problem of order 1–6 in Table5); **b**. evaluation of each chapter through SFCs

As per the survey of the 94 students (who participated in the SFCs), most respondents (71 vs. 23) considered SFCs as an interesting and attractive teaching method. Moreover, many students (70 vs. 24) agreed that SFCs cover a wealth of course content and lecturers’ presentations are clear and focused. Additionally, the SFCs not only helped students to summarise and memorise fragmented knowledge (83 vs. 11) but also enlightened extended learning (62 vs. 32). Furthermore, an overwhelming majority (93 vs. 1) of the respondents considered the SFCs as a link between theory and clinical practice (Fig. 4, Table 5).

**Table 5:** Student satisfaction regarding the semi-flipped classes

	Strongly agree	Agree	Neutral	Disagree
**Overall evaluation**				
1. The semi-flipped classes are interesting and attractive	59	12	23	
2. A pile of teaching content was present	42	28	24	
3. The lectures clearly explain difficult points	46	24	22	2
4. The video course summarises the textbook knowledge	72	11	11	
5. It is enlightening for in-depth study	30	32	30	2
6. It links theory with clinic practice tightly	68	25	1	
**Evaluation of each chapter**				
7. The chapter ‘oral bullous disease’ helped me to quickly seek common clinical features while reserving differences	60	21	13	
8. The chapter ‘oral allergic disease’ helped me to memorise knowledge points efficiently and form knowledge networks	61	26	7	
9. The chapter ‘oral ulcerous diseases’ helped me to extend textbook knowledge	66	20	8	
10. The chapter ‘oral patches stria diseases’ contributed significantly to enlivening the classroom atmosphere and increase teacher–student interaction	58	23	13	
11. The chapter “lip and tongue disease” helped me to learn about an abstract disease, burning mouth syndrome, which has no visible mucosal lesions	63	26	5	
12. The chapter ‘introduction of clinical thinking’ had instructive value in clinical practice	70	23	1	

### Evaluation of each chapter

According to the results of the questionnaire, each chapter achieved the expected teaching effect (Fig. 4, Table 5). The chapter ‘oral bullous disease’ helped students quickly seek common clinical features while preserving differences; ‘oral ulcerous diseases’ helped in extending textbook knowledge; and ‘oral patches stria diseases’ made a significant contribution to enlivening the classroom atmosphere and increasing teacher–student interaction. The SFCs broke through the barriers of textbooks and clinics and helped students understand the disease before clinical practice.

### Post-course assessment for semi-flipped class

**Fig. 5:**
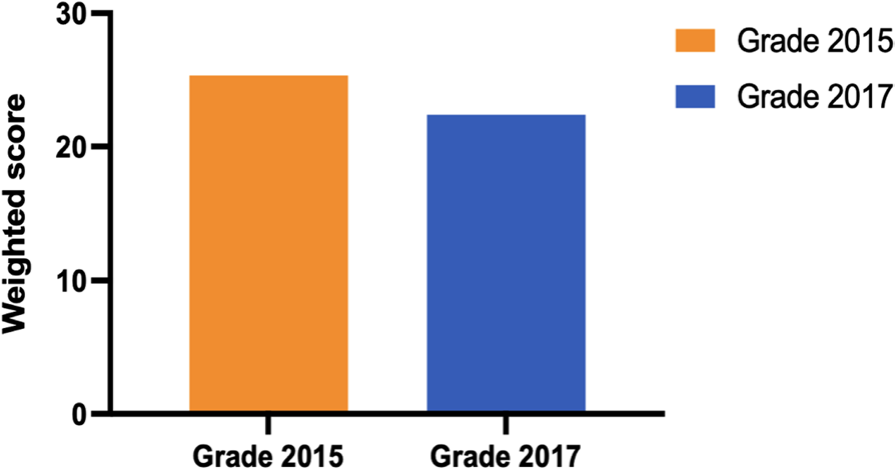
Comparison of final assessments of teaching reform group and control group. (*P* < 0.01)

DFs and SCs were determined as previously mentioned. After this step of homogenisation, we compared the exam results of the teaching reform group (grade 2015, *n* = 94) with those of the control group (grade 2017, *n* = 86). The results showed that the SFCs had a significant impact on improving learning outcomes, weighted score of 25.33(Grade 2015) vs. 22.39(Grade 2017) (*P* < 0.01) (Fig. 5).

## Discussion

With the development of online education, a variety of online learning models have emerged, such as MOOC and other massive course sharing or trading platforms; this has attracted the attention of medical educators. Pei et al. [19] conducted a meta-analysis of the literature in 2000–2017. They systematically reviewed 3700 articles, selected 16 articles that met the research standards, and discussed the degree of knowledge and skills mastered through online and offline learning for medical undergraduates. Compared with traditional offline learning, online learning is more effective in promoting the knowledge and skills of medical undergraduates; thus, it is a feasible method. Coincidentally, scholars such as Tang [20] reviewed 45 studies and concluded that the integration of online courses into medical education has a high degree of acceptance and provides satisfying learning results for medical undergraduates.

Generally, early online educational methods adopted the recording and broadcasting class model. Compared with our online education, they did not strictly require teachers to return to the classroom, and they did not have dual screens to enable the teacher to see the students [21]. Actually, online learning that consisted mainly of static, non-interactive learning resources is largely resembled offline learning [19]. This one-way video courseware cannot form a complete learning loop, leading to problems such as students’ absence, teachers’ self-talk, and lack of teacher–student interaction. Our results also showed that maintaining the interactions between teachers and students is only difficult in online courses. This is similar to the results of some previous studies. Among the three teaching methods, no teacher chose a purely online teaching mode. This is because they observed that students found difficulty in adapting to this teaching method, and their expressiveness and passion were lower than that in offline courses. A study summarized several key factors. The teachers found that the effectiveness of online learning is influenced by administrative issues, social interaction, academic skills, technical skills, learner motivation, time and support for studies, technical problems, cost and access to the internet [22]. All these factors could result in low-quality online learning. Therefore, online education alone should not be regarded as the most superior method. Compared with recording and broadcasting, live teaching is a more interactive method, which can create a better learning atmosphere and address the issue of mandatory learning to a certain extent [21]. According to the survey results of this study, as well as those of a recent study [23], traditional face-to-face education is irreplaceable for medical courses.

In contrast to purely online educational methods, our online educational method combines live broadcast technology and online education, which appeals strongly to the internet generation and compensates for the lack of online learning interaction [24]. This creates opportunities for the online education industry, which as a result, promotes the rise of live broadcast education. However, medical education is complex as it includes operational and humanistic concerns, which require teachers and students to interact sufficiently. Online and offline educational methods have their own advantages; however, combining them creates many problems, such as reduced classroom time and increased complexity of knowledge, which makes achieving satisfactory results difficult.

Additionally, undergraduate oral medical courses emphasized mainly on basic knowledge and some specifically skills [19]. In this study we successfully implemented SFCs and reformed the oral medicine curriculum of a college. The application of SFCs in oral medicine courses received satisfactory evaluations from learners and achieved satisfactory outcomes. Students (in grade 2015) who participated in the SFCs had significantly higher scores on their final exam than the control group (grade 2017). These results were similar to those based on the flipped-classroom courses analysed by other researchers [25, 26]; this might be owing to the great benefits of flipped classrooms. Through Huang’s study, thay just apply the flipped classes to the case study “reducing the hemolysis rate”, and three clinical cases were discussed during class lasted for about 30 min per session [25]. We not only provided clinical cases but also offered some related questions. When students participated in the classroom, the teacher could play a better guiding role through our class design. In our study, the improved teaching results were not only evident in the visible exam score but also in the positive evaluations on the reform of teaching methods (Fig. 4). SFC methods encourage students to obtain new concepts and information in advance, which is more effective than offline and online learning for some target knowledge and skills [19]. Thus, students will be equipped with factual knowledge to understand concepts and search for references and information. Through the SFC teaching method, students apply metacognitive strategies by self-learning [25]. For example, in this study, students who attended the SFCs chose a case and tried to make a diagnosis or solve some problems by themselves before class, rather than passively get the answers in traditional online or offline classes. Furthermore, implementing the SFC method in teaching oral medicine allowed students to develop critical thinking, analyse medical evidence rationally, and apply their knowledge to real clinical care practically. In the chapter ‘oral ulcerous diseases’, students strongly agreed that SFCs helped them understand new concepts and information in advance and helped extend their textbook knowledge. Most students mentioned that SFCs benefited their learning process. And this kind of activities were well related to the learning objectives of the class [19].

Nonetheless, designing an SFC is not an easy task [27]. During the course preparation process, educators may spend more time developing the knowledge points, collecting course materials, designing teaching methods, and recording video clips than they would for traditional offline classes. Some flipped classes study, teachers provided video or eBook [25, 27]. In contrast to fully flipped classes, the SFCs allowed educators to impart some knowledge during class time owing to the characteristics of oral medicine courses. Educators provided clear and detailed video clips to students so that they may learn before class; the educators used the classroom time to explain difficult points to inspire students to think independently and encourage interaction. Students were required to use the online courses, which entailed watching video clips and finishing the relevant exercises, to conduct self-learning before the class. This allowed them to attend class with some basic knowledge and specific questions. A meta-analysis found that an explanation for the more positive student perception, as well as the greater effect of flipped classroom over traditional classroom, is that students could watch the videos multiple times to better understand a particular topic [28]. In the classroom, not only do students learn the curriculum content from the educators but also the methods to study medical science efficiently and solve real problems in clinical practice. However, students also report that watching video before class have increased the hours devoted to the course. Especially in a high academic load semester, this can result in the inclusion of an additional stress factor [29].

Though the SFC educational method showed positive results, this study had some limitations. First, the experimental design was not ideal as many factors could not be set randomly, such as the participants, educational environment, and learning abilities. Second, the number of participants in this study was low. Future applications of this teaching method in larger groups are needed to determine whether the results achieved in this study are generally consistent. Third, our course contained only six classes, which was not enough to predict long-term training performance. Therefore, more studies are needed to track the long-term learning effect and determine whether the beneficial outcomes of SFCs are sustainable.

## Conclusions

According to our surveys, teachers and students believe that online education is an extension of traditional offline education. Effective and appropriate application may help further improve the teaching effect; however, fully replacing traditional offline education is not possible. We planned to promote online education and use online, offline, intracurricular, and extracurricular teaching modes.

The semi-flipped and interactive class design will play a pivotal role in encouraging teachers to enrich classroom teaching content and in inspiring teaching reform. Teachers can make full use of this video platform to flip the classroom, thereby turning curricular teaching into extracurricular or even off-campus learning. This change will greatly improve traditional teaching methods and expand students' thinking, which will enable them to learn actively. Additionally, it can cultivate students' abilities to think innovatively and practically through various forms of exchanges and cooperation, such as group discussions and debate competitions.

The question of how to transfer more knowledge quickly and efficiently to medical students and invite them to think independently is a huge challenge for educators. Our SFCs achieved teaching reform successfully and built a platform for both educators and learners so that teaching and learning support each other. However, further work on curriculum reform is still required (for instance, a long-term and larger group study and a study involving a public open course or MOOC) to increase knowledge attainment further and achieve deeper multidisciplinary integration.

## Data Availability

The datasets used and/or analysed during the current study are available from the corresponding author on reasonable request. We do not have ethical permission to upload the dataset into a repository. Please note that all study data has been anonymised for confidentiality purposes.

## Abbreviations

MOOC: Massive open online course
SFC: Semi-flipped class
OS: Original score
DF: Difficulty factor
SS: Standard score
